# Nutritional and Quality Characteristics of Some Foods Fortified with Dried Mushroom Powder as a Source of Vitamin D

**DOI:** 10.1155/2022/2792084

**Published:** 2022-07-26

**Authors:** Rehab Mohamed Ibrahim, Maha IK Ali, Faten Farouk Abdel-salam

**Affiliations:** ^1^Department of Special Food and Nutrition Research, Food Technology Research Institute, Agricultural Research Center, Giza, Egypt; ^2^Department of Food Science and Technology, Faculty of Agriculture (El-Shatby), Alexandria University, 21545 Alexandria, Egypt

## Abstract

Vitamin D plays a vital role in synthesizing calcium-carrying proteins in the small intestine and helps the absorption of calcium in the body, thus reducing the risk of rickets in children and osteoporosis in adults, especially in women. So, the objective of this study was to evaluate the nutritional value and quality characteristics of some food products such as waffles, breadsticks and salad cream fortified with dried mushroom powder (DMP) after exposure to sunlight for 60 min as a source of vitamin D. The exposure of mushroom to sunlight for 60 min before drying increased its content of vitamin D by 158% more than fresh mushroom (not exposed to sunlight). The DMP was added to the product's formula by a ratio of 1, 2, and 3%. The addition of DMP increased protein, ash, fat, and vitamin D_2_ and D_3_ contents in all products, while carbohydrates and moisture contents were decreased in both waffles, and breadsticks. The hardness of both waffles and breadsticks was decreased with increasing the levels of DMP added, while the addition of DMP led to enhance bioactive compounds and antioxidant activity in all products. The sensory evaluation of waffles, breadsticks, and salad cream containing DMP was not changed than control sample. The results found that the intake of 100 g of salad cream, waffles, and breadstick (containing 3% DMP) could by providing more than the recommended daily allowances (RDA) of vitamin D. Therefore, this study recommended the use of DMP (by a ratio of 3%) in fortifying food products in order to meet the RDA of vitamin D.

## 1. Introduction

Vitamin D is one of the micronutrients that have a unique position in many aspects. It is essential for bone, muscle growth and development in children. It can be synthesized in the body through direct exposure to sunlight, which is why it is considered a hormone [1, 2]. Vitamin D is a very limited source in food and therefore does not meet the needs of the body from this vitamin, so vitamin D deficiency has become one of the most important problems prevailing at the global level [3, 4].

Vitamin D plays an important role in the synthesis of calcium-carrying proteins in the small intestine, works on the nutritional absorption of calcium, and thus reduces the risk of rickets in children and osteoporosis in adults. It also helps prevent some cancers, respiratory diseases, cardiovascular diseases, degenerative diseases, neurological diseases, and type 1 and 2 diabetrs [5, 6]. Vitamin D is a hormone consisting of a group of the fat-soluble mixture of steroid derivatives whose main function is to increase the absorption of calcium and phosphate from the intestines. There are two forms of vitamin D, vitamin D_3_ (cholecalciferol) and vitamin D_2_ (ergocalciferol). It was found that 90% of vitamin D in the body is vitamin D_3_ (cholecalciferol) made by the skin, and 10% is vitamin D_2_ (ergocalciferol) ingested from the diet [7, 8]. Few foods are a good source of vitamin D, and fish oils are one of the best sources of it. Currently, some countries have policies on fortifying foods with vitamin D, such as milk, margarine, breakfast cereals, and juices [9, 10].

Several studies have indicated that the recommended daily allowances (RDA) for vitamin D varies with age. The RDA of children aged 1–8 y is 600 IU/d or 15 *μ*g/day. For men and women aged from 9 to 70 y, and pregnancy and lactation, the RDA is 600 IU/day or 15 *μ*g/day. For men and women aged from >70 y, the RDA is 800 IU/day or 20 *μ*g/day [11, 12].

Mushroom is considered a gourmet food all over the world because of its unique flavor. It is a functional food of high nutritional value. Furthermore, it has a great interest due to its organoleptic advantages, medicinal characteristics, and high economic importance [13, 14]. Mushroom are a low-calorie diet that approximately ranged from 250 to 350 calories/kg of fresh mushroom because it has low-fat content (2-6%) and also contains protein ranging from 19 to 35% and 50 to 65% total carbohydrates [15]. In addition, mushroom contain all the essential amino acids such as lysine and tryptophan, as well as aspartic acid, glutamic acid, and arginine which makes mushroom an excellent alternative to animal products [16, 17]. As well as, it contains large amounts of vitamins, such as B_1_, B_2_, B_3_, B_5_, B_6_, B_7_, B_12_, and vitamin D, and minerals such as potassium (K), calcium (Ca), magnesium (Mg), phosphorus (P), selenium (Se), and copper (Cu) [16].

Mushroom are now considered a good source of antioxidants, anti-inflammatory, immunostimulatory, antimicrobial, and hypocholesterolemic compounds such as ascorbic acid, ergosterol, phenolic compounds, tocopherols, carotenoids, and dietary fibers, where it can provide 25% of the recommended daily intake of dietary fiber [15, 17]. Mushroom, like the human skin, can produce vitamin D when exposed to sunlight. During this process, ergosterol is converted to ergocalciferol (vitamin D_2_) within a series of photochemical and thermal reactions [18]. The amount of vitamin D that is made in mushroom is considerably higher than the content of vitamin D in fortified products of the food industry. Several studies have indicated that the deliberate exposure of mushroom to sunlight in the middle of the day for 15-120 min would generate large amounts of vitamin D, which usually exceeds 10 *μ*g/100 g FW, and this rate is close to the recommended daily allowances of vitamin D in many countries. In Germany, it was found that the vitamin D content in mushroom slices exposed to sunlight was as high as 17.5 *μ*g/100 g FW after 15 min of sun exposure and reached 32.5 *μ*g/100 g FW after 60 min of sun exposure [19–21]. Therefore, the aim of this study was to utilize the dehydrated mushroom powder after exposure to sunlight as a source of vitamin D in preparing different food products such as waffles, breadsticks, and salad cream.

## 2. Materials and Methods

### 2.1. Materials

Fresh oyster mushroom (*Pleurotus ostreatus*) was obtained from Plant Protection Research Station, Plant Pathology Research Institute, Agriculture Research Center (El-Sabahia), Alexandria, Egypt. Other ingredients including eggs, wheat flour (72% extraction), sugar, dry yeast, spices (cumin, white pepper, and onion powder), vinegar, corn oil, milk, vanilla, salt, and plastic bag were obtained from Alexandria markets, Egypt. 1,1-Diphenyl-2-picrylhydrazyl (DPPH) was purchased from Sigma-Aldrich (Munich, Germany). Ferric chloride, potassium ferricyanide, sodium nitrite, aluminum chloride, trichloroacetic acid (TCA), rutin, and gallic acid were obtained from Loba Chemie, Mumbai, India.

### 2.2. Methods

#### 2.2.1. Technological Method

*(1) Preparation of Mushroom*.

- *Fresh Mushroom Processing Method*. The fresh oyster mushroom (*Pleurotus ostreatus*) was divided into three parts. The first part has not been exposed to the sunlight, while the second and third parts were deliberately exposed to sunlight for 60 and 120 min (from 9 to 11 am) during October 2020 (the average temperature was 21°C), in order to study the effect of exposure to sunlight on the formation and concentration of vitamin D in mushroom samples
- *Preparation of Dehydrated Mushroom Powder*. Ten kilograms of fresh mushroom was cleaned (sorting and trimming); then, it is exposed to sunlight for 60 min (according to the HPLC results of vitamin D), after that it was cut into 2 mm thickness slices, then dipped in citric acid (0.5%) solution for 30 min, then drained again and spread in a single layer on stainless steel trays and dehydrated at 45°C for 12 hours in a thermostatically controlled hot air oven (Vacuum Oven Model 3618, USA). The dehydrated mushroom powder (DMP) was obtained by electrically grinding (Moulinex, AR1044) dehydrated slices to pass through 80 mesh sieves. The obtained flour was packed in an air-tight Kilner jar and kept at 4°C until used

*(2) Preparation of Waffles*. The waffles were prepared based on the method of Kigozi et al. [22]. The waffles formula is illustrated in Table 1, where the wheat flour in the formula was replaced with 1, 2, and 3% (*W*/*W*) DMP. All ingredients were mixed well using a blender (Kenwood major titanium, Japan); then, mixtures were lifted for a few minutes before baking. The mixture (15 g) was poured into the waffles maker for 3.5 min at 150°C. Finally, it was stored in completely deflated bags to preserve the crispness of the waffles.

*(3) Preparation of Breadsticks*. Breadsticks were prepared according to El-Hadidy et al. [23]. The breadsticks formula is illustrated in Table 1. The wheat flour in the reference method was replaced by 1, 2, and 3% (*W*/*W*) DMP. The sugar and instant dry yeast were mixed into the warm water, and then, the flour was mixed with salt and oil. All ingredients were mixed well by hand until soft dough was formed. The dough was wrapped in a plastic bag and left to ferment for 30 min at room temperature (30 ± 2°C). The dough was divided into small pieces in the form of sticks and left to ferment for 30 min at 30°C, then transferred to ungreased baking sheets and baked in preheated oven at 150°C for 45 min.

*(4) Preparation of Salad Cream*. Salad cream was prepared using 1, 2, and 3% (*W*/*W*) of DMP based on the method of Babajide and Olatunde [24] with some modifications, and the formula of each treatment is illustrated in Table 1. On fire (70 ± 2°C), the flour was roasted for 1 min; then salt, sugar, corn oil, spices, liquid milk, vinegar, and water were added. All components were mixed well using a hand blender, and then, the mixtures were left on fire for 4 min with gently mixed, then cooled and packaged in glass jars.

#### 2.2.2. Analytical Methods

*(1) Chemical Analysis*.

- *Determination of Vitamins D_2_ and D_3_*. The vitamin D_2_ and D_3_ contents were determined according to the procedure of AOAC [25] using HPLC. The HPLC condition systems are as follows: controller: Shimadzu SCL 6A; oven: CTO-6A column; detector: SPD 6AV; pump: LC-6A liquid chromatography; column: ODS reversed-phase (octadodelylsulphate); UV-visible detector at oven 264 nm/25, with flow rate 1 mL/min.; injection volume 25 *µ*L; HPLC (Shimadzu 24); mobile phase: methanol: water 99 : 1; and elution: isocratic elution. Concentration of vitamin D in sample = (peak area of sample × concentration of standard)/peak area of standard. All analyses were performed in triplicate
- *Determination to Proximate Composition*. A proximate composition (moisture, protein, fat, ash, and crude fiber) was determined by AOAC [25]. Carbohydrates (NFE) were calculated by difference. All analyses were performed in triplicate
- *Determination of Minerals*. Calcium (Ca), sodium (Na), iron (Fe), potassium (K), zinc (Zn), magnesium (Mg), manganese (Mn), and phosphorus (P) were measured in ash solution using ICP-OES Agilent 5100 VDV based on the method of AOAC [25]. All analyses were performed in triplicate

*(2) Bioactive Compounds and Antioxidant Activity*.

(1) *Preparation of Ethanolic Extracts*. The ethanolic extracts of mushroom (not exposed and exposed to the sunlight), dried mushroom powder, and final products (waffles, breadsticks, and salad cream) were prepared based on the method of Öztürk et al. [26]. Five grams of each mushroom sample and final products was mixed with 30 mL ethanol (75%), stirring for 2 hours at room temperature, and then, filtered using Whatman No.1, and the extracts were stored at -20°C until analysis
(2) Determination of Total Phenolic Contents. Total phenolic contents (TPC) of extracts were determined in triplicate using the method developed by Abirami et al. [27]. Folin–Ciocalteu's reagent (1.5 mL, diluted 10 times) and Na_2_CO_3_ (1.2 mL, 7.5% *w*/*v*) were added to water-soluble extract (300 *μ*L), mixed well, and kept for 30 min at room temperature before measuring at 765 nm using a spectrophotometer (Pg T80+, England). Total phenolic contents were calculated as mg gallic acid equivalent per gram of plant material or extract
(3) Determination of Total Flavonoid. The total flavonoid content of extracts was determined in triplicate according to Barros et al. [28]. A half milliliter of each extract was mixed with 2 mL distilled water followed by 150 *μ*L of NaNO_2_ solution (5%). After 6 min, 150 *μ*L of AlCl_3_ (10% *w*/*v*) was added. The mixtures were left for 6 min at room temperature before the addition of 2 mL NaOH (4% *w*/*v*). The mixture was brought to 5 mL with distilled water and left at room temperature for 15 min before measuring at 510 nm using a spectrophotometer (Pg T80+, England). Total flavonoid was expressed as *μ*g of rutin equivalent/mL extract (*μ*g RE/mL)
(4) DPPH Scavenging Activity. The radical scavenging activity of 1,1-diphenyl-2-picrylhydrazyl (DPPH) was determined in triplicates according to the method of Brand-Williams et al. [29]. One milliliter of each extract was mixed with 2 mL DPPH solution (3 mg DPPH/100 mL methanol). The mixtures were left in the dark at room temperature for 30 min. Absorbance (Abs) was measured at 517 nm against a blank (distilled water) using a spectrophotometer (Pg T80+, England). Control was prepared by replacing the extract with methanol. The radical scavenging activity of extracts was calculated as follows:
  (1) $$\begin{array}{c} \text{scavenging activity}\%=\frac{\left(\text{ A control}-\text{A sample }\right)}{\text{Acontrol}}\times 100 \end{array}$$
(5) *Ferric Reducing Antioxidant Power (FRAP)*. FRAP values were determined according to the method of Oyaizu [30]. Two and half milliliters of 0.1 M phosphate buffer (pH 6.6) and 2.5 mL of 1% (*w*/*v*) potassium ferricyanide were added to 1 mL of each extract and then mixed well and incubated at 50°C for 20 min in a water bath, then cooling to room temperature. 2.5 mL TCA (10% *w*/*v*) was added to all tubes and then centrifuged at 10,000 × *g* for 10 min at 4°C. Next, 2.5 mL supernatant from each tube was mixed with distilled water (2.5 mL) and ferric chloride solution (0.5 mL, 0.1% *w*/*v*). After that, the mixtures were left in dark at room temperature for 30 min; then, the absorbance was measured at 700 nm using a UV/visible spectrophotometer, Pharmacia-LKB-Ultrospec III (Pharmacia, USA). The assay was done in triplicate. The FRAP values were derived from a standard curve as mg gallic acid equivalents (GAE/g)

### 2.3. Physicochemical Properties

#### 2.3.1. Color Measurement

Color values such as the *L*^∗^ (lightness), *a*^∗^ (red intensity), and *b*^∗^ (yellow intensity) of the final products were measured using a Hunter Lab Ultra Scan, VIS model, and colorimeter (USA). The instrument was standardized during each sample measurement with a black and white tail (*L*^∗^ = 94.1, *a*^∗^ = 1.12, *b*^∗^ = 1.26). The mean of five readings of each color index of the Hunter scale (*L*^∗^, *a*^∗^, *b*^∗^) was recorded [31].

### 2.4. Texture Profile Analysis (TPA)

Texture profile analysis of waffles, breadsticks, and salad cream was performed using TA-XT 2 Texture meter (Texture Pro CT3 V1.2, Brookfield, Middleboro, USA) based on the method of Yuan and Chang [32]. The curves of force-time deformation were obtained by applying a load of 5 kg at a 1 mm/s cross head speed. The texture parameters such as hardness (g), resilience, chewiness (mJ), gumminess (g), cohesiveness, and springiness (mm) were determined.

### 2.5. Sensory Evaluation of Products

Color, taste, odor, texture, and overall acceptability of waffles, breadsticks, and salad cream samples were assessed using 10 panelists (5 males and 5 females) from the Department of Food Science and Technology, Faculty of Agriculture, University of Alexandria. The panelists were asked to evaluate the above characteristics depending on a standard hedonic rating scale from 1 (dislike extremely) to 9 (like extremely) based on the method of Banach et al. [33].

### 2.6. Statistical Analysis

All data were analyzed by a general linear model procedure (GLM) using SAS statistical analysis software package [34]. The statistical analysis was performed using one-way ANOVA. Means were compared by Duncan's test at the significance level of *P* ≤ 0.05.

## 3. Results and Discussion

### 3.1. Effect of Exposure to Sunlight on the Formation of Vitamins D_2_ and D_3_ in Mushroom

**Figure 1:**
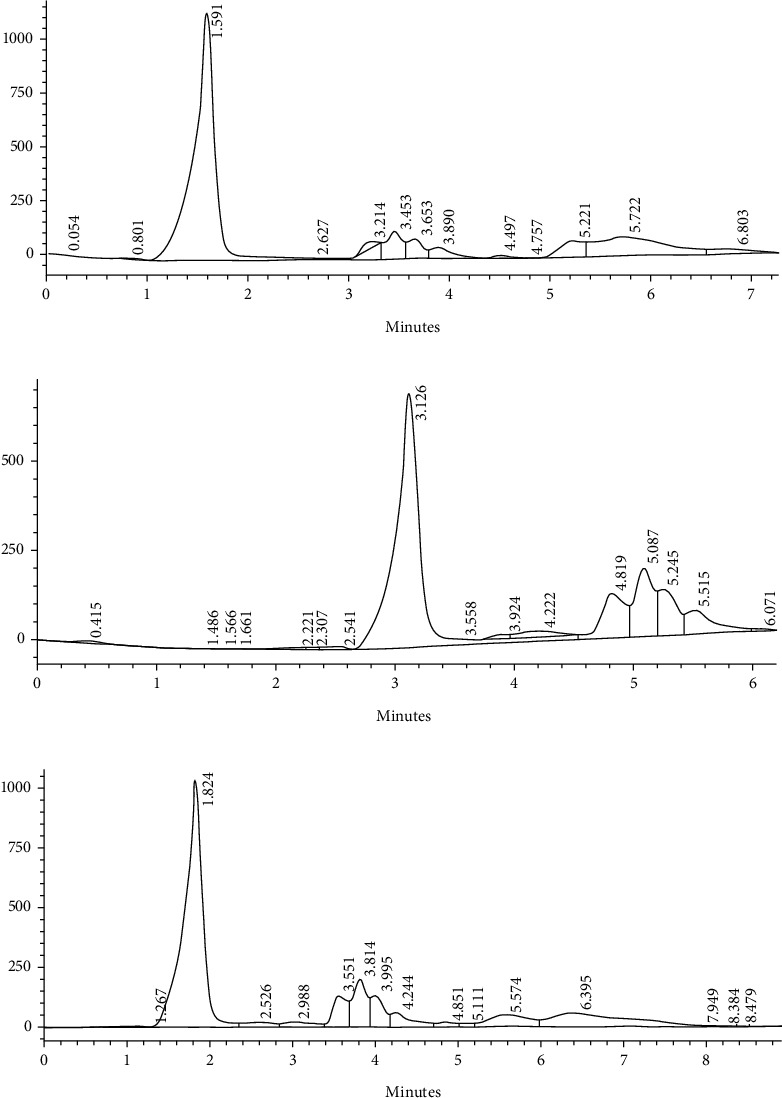
Effect of exposure to sunlight on the formation of vitamins D_2_ and D_3_ in fresh mushroom: (a) not exposed, (b) exposed to sunlight (60 min), and (c) exposed to sunlight (120 min).

The content of V.D in fresh mushroom exposed and not exposed to sunlight are shown in Table 2 and Figure 1. The results indicated that the significantly (*P* < 0.05) highest content of vitamin D_2_ was found after the exposure of fresh mushroom to sunlight for 60 min (77.61 *μ*g/100 g fresh weight) followed by the fresh mushroom exposed to sunlight for 120 min (44.61 *μ*g/100 g fresh weight). Meanwhile, the lowest content of vitamin D_2_ was observed with fresh mushroom samples that were not exposed to sunlight (30.03 *μ*g/100 g fresh weight). On the contrary, vitamin D_3_ content in by fresh mushroom exposed to sunlight for 120 min (99.54 *μ*g/100 g fresh weight) was significantly (*P* < 0.05) higher than the fresh mushroom exposed to sunlight for 60 min (70.80 *μ*g/100 g fresh weight) and fresh mushroom not exposed to sunlight (62.81 *μ*g/100 g fresh weight). The obtained results in this study are similar mostly to those reported by Cardwell et al. [35] who mentioned that deliberate exposure of mushroom to sunlight in the middle of the day for 15-120 min generates large amounts of vitamin D_2_, often in excess of 10 *μ*g/100 g fresh weight. Moreover, the amount of vitamin produced depends on the time of day, season, latitude, weather, conditions, and exposure time. Also, these findings are in line with those reported by Urbain and Jakobsen [21] who found that sunlight exposure (for 60 min) increased the vitamin D_2_ content of the mushroom from 0.1 *μ*g/g up to 3.9 ± 0.8 *μ*g/g dry weight (DW) of fresh mushroom and these values comparable to levels found in fatty fish like the Atlantic salmon. Phillips and Rasor [20] observed that the vitamin D_2_ content in mushroom exposed to sunlight for 30 min was 36.6 *μ*g/100 g fresh weight. Also, Cardwell et al. [35] found that mushroom contains vitamin D_2_ above 10 *μ*g/100 g fresh weight, which is higher than the level in most foods vitamin D containing and equal to the daily allowances of vitamin D recommended internationally. Consequently, its consumption has increased in recent decades, as mushroom are an important source of vitamin D_2_ from a non-animal source. Also, Simon et al. [36] stated that there was no significant difference in vitamin D content between the mushroom exposed to UVB light (5.80 mg/g) and control (5.78 mg/g), while the exposure to sunlight led to a significant increase in vitamin D content (6.33 mg/g).

### 3.2. Chemical Composition, Bioactive Compounds, and Antioxidant Activity of Fresh Oyster Mushroom (Not Exposed and Exposed to Sunlight for 60 min)

Mushroom have many good benefits for the human diet: they are high in protein and dietary fiber and low in fat, as well as being good sources of vitamins, minerals, and nutraceuticals [37, 38].  Table 3 shows the chemical composition of fresh mushroom (not exposed and exposed to sunlight). The results indicated that there is no significant (*P* > 0.05) differences in moisture, protein, fat, ash, fiber, and NFE contents among the fresh and exposed to sunlight mushroom. According to the study of Prodhan et al. [39], the fresh mushroom contained more than 87% moisture, 5.14% protein, 2.75% fiber, and 1.40% ash, while our study found that the contents of moisture, protein, fiber, and ash in fresh mushroom was 91.13, 2.37, 0.560, and 0.459, respectively. Generally, the nutritional composition of mushroom differs considerably depending on many factors such as species, interspecies genetic variability, growth conditions, maturity, environmental conditions, geographic location, and postharvest conditions [40, 41]. The results also revealed that the fresh mushroom not exposed and exposed to sunlight (60 min) contained 5.28 and 7.00 mg GAE/g total phenolic content, respectively, while the total flavonoid content in mushroom not exposed and exposed to sunlight were 6.90 and 18.26 *μ*g/mL, respectively. The mushroom sample exposed to sunlight showed higher DPPH scavenging activity (78.74%) compared to not exposed samples (65.88%). Also, a higher FRAP value was observed with mushroom samples exposed to sunlight (Table 3). These obtained values are higher than those found by Azieana et al. [42] who reported that the total phenolic content of fresh mushroom was 0.024 mg GAE/g.

### 3.3. Chemical Composition, Mineral Content, Vitamin D Content, Bioactive Compounds, and Antioxidant Activity of Dry Mushroom Powder (DMP) Exposed to Sunlight (60 min)

Table 4 illustrates the moisture, protein, fat, ash, fiber, and NFE values (11.13%, 26.71%, 1.84%, 5.17%, 5.19%, and 49.96%, respectively) in DMP. These values of moisture, fat, fiber, and carbohydrate were less than those obtained by Das et al. [43], while protein and ash values were higher. This may be due to the difference in drying technique and final moisture content. Prodhan et al. [39] noted that mushroom powder was richer in crude protein, crude fiber, and ash as compared to fresh mushroom. The average of protein content in mushroom powder was 24.51% protein, 12.00% crude fiber, and 5.54% ash.

The results showed that the content of DMP from Na, K, Ca, Fe, Zn, P, Mn, and Mg was 239.08, 1213.02, 106.41, 6.98, 13.27, 912.50, 2.43, and 13.18 mg/100 g, respectively (Table 4), while Igile et al. [44] found that the mineral elements like Ca, Fe, and K in oyster mushroom were 32.17, 56.44, and 1085.09 mg/100 g, respectively. These results are in agreement with Gençcelep [45] who reported that mushroom had the highest content in some minerals such as iron, manganese, copper, and zinc which play an essential role in the proper functioning of various metabolic paths. Moreover, the mushroom contained higher amounts of some important trace elements such as phosphorus and potassium compared with most vegetables. Our study also noted that the concentrations of Ca and Zn in DMP were higher than the finding by Siyame et al. [46] who found that the DMP contained 8.3 and 49.45 mg/100 g of Zn and Ca, respectively. Meanwhile, the Fe and P concentrations were the lowest.

The data in Table 4 cleared that the contents of vitamins D_2_ and D_3_ in the dried mushroom powder were 889 and 811 *μ*g/100 g, respectively. These values were higher than those reported by Rangel-Castro et al. [47] who found that the content of vitamin D_2_ in dried mushroom ranged from 12 to 630 *μ*g/100 g. These results may be due to the different variety, climatic conditions, and drying methods used. It could be noted that the total phenolic, total flavonoid, DPPH scavenging activity, and FRAP values in dried oyster mushroom were 6.02 mg GAE/g, 86.29 *μ*g/mL, 90.45%, and 0.420 mg GAE/g, respectively. This may be due to that treatment with citric acid before drying leads to an increase in the level of total phenolic and total flavonoid [48]. These values are higher than those reported by Babu et al. [49] who found that the total flavonoid and total phenolic content in oyster mushroom ranged from 1.2 to 2.9 *μ*g/g and ~ 2 to >30 mg/g, respectively. It is worth noting that phenolic compounds are mushroom antioxidants which are strong radical scavengers and free radical inhibitors and phytonutrients [50].

### 3.4. Chemical Composition of Waffles, Breadsticks, and Salad Cream

Enriching or fortifying food products with a good source of protein containing all the essential amino acids may support reducing the incidences of protein-energy malnutrition in humans [51]. The earlier studies demonstrated that mushroom rich in protein possess all nine amino acids that are essential for humans and are excellent sources of dietary fiber, while it was low in fat and calories [52]. The chemical composition of the waffles was presented in Table 5. It could be noticed that the addition of DMP in the components of waffles significantly (*P* < 0.05) increased protein and ash contents which ranged from 4.50% to 5.74% and 0.69% to 0.73%, respectively. The highest protein content was observed in the W3 sample whereas the lowest values were found in W0 and W1 samples.

Results also indicated that there were no significant differences in the moisture, fat, and NFE contents among all treatments. The obtained contents of protein, fat, ash, and fiber were less than those obtained by Farooq et al. [53] who found that fortifying the muffin with mushroom powder increased the protein content from 9.45 to 9.59%, fiber from 0.30 to 0.35%, fat from 1.25 to 1.31%, and ash from 0.35 to 0.41%. The increase of ash content in waffles may be attributed to the DMP which consider a good source of minerals like phosphorus, potassium, iron, copper, and zinc [54], whereas the increase in protein content could be attributed to the addition of mushroom which is rich in protein content and to other components like an egg [17, 55].

The chemical composition of breadsticks is shown in Table 5. It was observed that by increasing the substitution level of DMP in the breadsticks formula, the moisture content decreased. The moisture content was in ranged from 28.37% (BS0 sample) to 27.26% (BS3 sample). Protein content was increased with increasing DMP levels. The higher protein and ash contents were found with BS3 (16.22% and 2.57%, respectively). Results indicated that there was no significant effect of DMP addition on fat and NFE contents of all breadsticks treatments. These findings are in agreement with those reported by Losoya-Sifuentes et al. [56], where adding mushroom flour to bread increased moisture, ash, fat, and protein contents.

Data in Table 5 illustrated the chemical composition of salad cream. It was found that moisture, fat, and protein contents were increased with increasing DMP levels in salad cream. No significant differences were observed in moisture and fat contents between all treatments. On the other hand, higher contents of protein and ash were found with SC3 sample (3% DMP), while the higher content of NFE was observed with SC0 sample. This may be due to the reduction of the amount of wheat flour used in preparing the formulas which are accompanied by reducing the protein content, but the mushroom flour contributed to maintaining the increase in protein despite the decrease in wheat flour. Furthermore, the observed substitution level of DMP increased significantly (*P* < 0.05) in ash content from 1.50% in the SC0 sample to 2.45% in the SC3 sample. On the contrary, the NFE content was decreased with the increase in DMP level in the salad cream formula. No significant difference was observed in NFE content among all treatments except with salad cream containing 3% DMP (SC3). These results are in agreement with those found by Srivastava et al. [57] who stated that the dried oyster mushroom powder incorporation into instant soup premix increased protein, ash, and crude fiber contents, however decreased carbohydrate, moisture, and fat contents.

### 3.5. Vitamin D_2_ and D_3_ Contents in Different Products

The contents of vitamin D_2_ and D_3_ in the waffles, breadsticks, and salad cream products are shown in Table 6. Results indicated that the addition of DMP increased significantly (*P* < 0.05) the vitamin D_2_ and D_3_ contents in the waffles from 0.995 to 15.458 *μ*g/100 g and 1.48 to 12.47 *μ*g/100 g, respectively. Moreover, the highest (*P* < 0.05) content of vitamin D was found in the W3 sample. Meanwhile, the W0 recorded the lowest contents of vitamins D_2_ and D_3_. These findings are due to the higher content of vitamin D_2_ in mushroom powder. It could be noticed that increasing the substitution level of DMP in breadsticks led to increase in vitamin D_2_ content from 0.569 *μ*g/100 g in BS0 to 12.35 *μ*g/100 g in BS3. Likewise, vitamin D_3_ content increased significantly from 0.42 *μ*g/100 g in BS0 to 9.168 *μ*g/100 g in BS3.

In salad cream, the vitamin D_2_ content ranged from 0.55 to 18.12 *μ*g/100 g. The highest value of V.D_2_ was found in SC3 sample, whereas the lowest value was found in SC0 sample. Moreover, the vitamin D_3_ content ranged between 0.608 and 13.647 *μ*g/100 g, and the highest (*P* < 0.05) content was found in SC3 sample, while the lowest (*P* < 0.05) content was observed in SC0 sample.

Generally, adding the dried mushroom powder to products helped increased significantly vitamin D_2_ and D_3_ contents in all products. These results are due to the fact that mushroom powder contains high concentrations of vitamin D_2_. Studies have indicated that mushroom are considered a vital source of vitamins, as they contain large amounts of vitamin D_2_ [15]. Moreover, edible mushroom are excellent sources of vitamin D that range from 4.7 to 194 mg/100 g [58].

The data in Table 6 showed the estimation of vitamin D intake for all products. The results revealed that according to the recommended daily allowances of vitamin D, it was found that the waffles (100 g) can cover 6.63–103.06% and 9.87-83.15% of the recommended daily allowances of vitamins D_2_ and D_3_, respectively, for children (1-8 Y), males and females (9-70 Y), and pregnancy and lactation. While in males and females (> 70 Y), the waffles can cover 4.97–77.29% and 7.4-62.36% of the RDA of D_2_ and D_3_, respectively. Concerning the breadsticks, it was found that 100 g of breadsticks can cover 3.79–82.35% and 2.81–61.12% of the RDA of vitamins D_2_ and D_3_, respectively, for children (1-8 Y), males and females (9-70 Y), and pregnancy and lactation, and 2.84–61.76% and 2.1–45.84%, respectively, for males and females (>70 Y).

Regarding the salad cream, it was found that 100 g can cover 3.66–120.8% and 4.05–90.98% of the RDA of vitamins D_2_ and D_3_, respectively, for children (1-8 Y), males and females (9-70 Y), and pregnancy and lactation, and 2.75–90.61% and 3.03–68.23%, respectively, for males and females (>70 Y). Several studies have indicated that the RDA of vitamin D between countries vary depending on the age. According to a recent report of the Institute of Medicine, the RDA for vitamin D should be 15 *μ*g/day for children greater than 1 year old and adults and 20 *μ*g/day for the elderly. As such, there is a renewed interest in improving dietary intake to meet these recommendations [11, 59]. According to the study by Kumar et al. [60] on young children in the US, it was that 50% of children 1–5 years old and 70% of children 6–11 years old had vitamin D deficiency. These findings are similar to that has been observed in New Zealand, Canada, Australia, Brazil, India, Mongolia, Africa, and the Middle East where more than 90% of both children and adults have been reported to be deficient or insufficient in vitamin D [1, 61–64]. In this study, it was found that the intake of 100 g of waffles sample containing 3% of DMP could be providing all RDA of vitamin D_2_ and most RDA of vitamin D_3_, while the intake of 100 g of salad cream sample containing 3% of DMP could be providing more than RDA of vitamin D_2_ and most RDA of vitamin D_3_. The major cause of the vitamin D deficiency is due to the lack of that foods naturally contain it. Meanwhile, most children and adults have always depended on sun exposure to get their vitamin D requirements [1]. Despite being cultivated in the dark and being a non-animal food source, mushroom contain high levels of vitamin D, which is often referred to as the sunshine vitamin. Upon exposure to sunlight or ultraviolet (UV), the vitamin D content (especially vitamin D_2_) of mushroom increases appreciably, which can play a significant role in the bone and cartilage health of vegetarians [14].

### 3.6. Bioactive Compounds and Antioxidant Activity of Waffles, Breadsticks, and Salad Cream

Recent research has focused on the importance of biologically active compounds in enhancing the health benefits of foods, so there is a great benefit of using mushroom as a food ingredient to improve the nutritional value of foods. It is a natural source of antioxidants, anti-inflammatory, anticancer, and anticolitis properties so there is interest to incorporate mushroom into food [65, 66].

**Figure 2:**
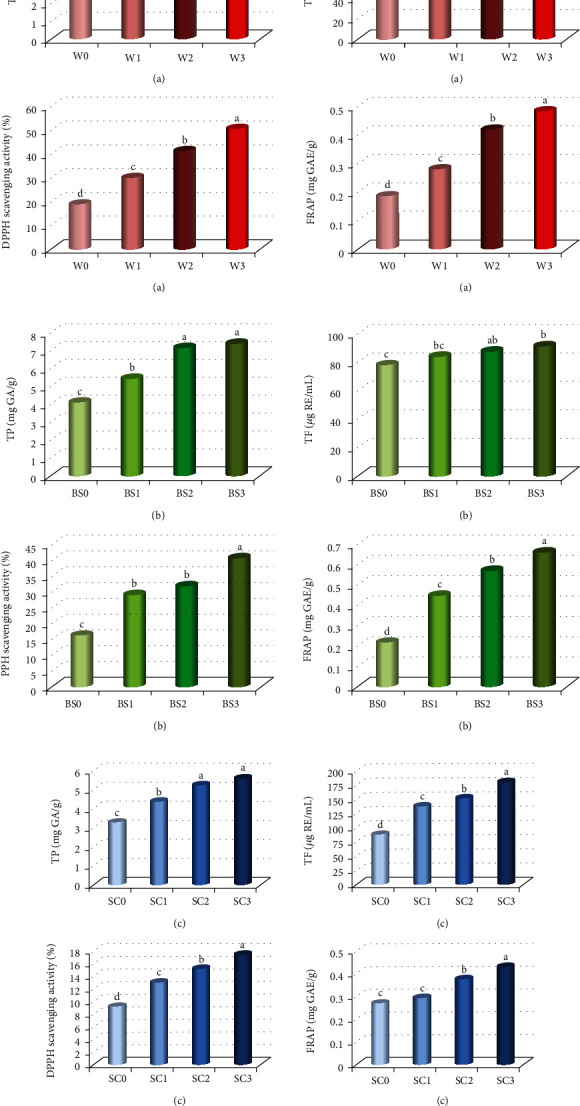
Bioactive compounds and antioxidant activity of (a) waffles, (b) breadsticks, and (c) salad cream. TP: total phenolic; TF: total flavonoid; RE: rutin equivalent; GAE: gallic acid equivalent; FRAP: ferric reducing antioxidant power; DPPH: 1,1-diphenyl-2-picrylhydrazyl.

Total phenolic (TP), total flavonoid (TF), DPPH scavenging activity (%), and FRAP values of waffles, breadsticks, and salad cream samples are illustrated in Figure 2. It could be observed that increasing levels of DMP in the waffle formula led to increasing the TP content from 5.63 mg GA/g in W0 to 7.33 mg GA/g in W3. As well as, the TF increased from 69.61 to 130.02 *μ*g/mL in W0 and W3, respectively. Moreover, the addition of DMP caused significant increase in DPPH scavenging activity (%) and FRAP values in waffles samples extracts. The higher DPPH scavenging activity and FRAP values were obtained with W3 sample while the lowest values were found with W0 sample (Figure 2(a)).

Also, it was observed that by increasing the substitution level of DMP in breadsticks, the TP content increased, and the higher TP content was found with BS3 (7.41 mg GAE/g), while the BS0 (4.09 mg GA/g) was the lowest. Likewise, the TF increased from 78.28 *μ*g/mL in the BS0 sample to 91.49 *μ*g/mL in the BS3 sample. Also, higher DPPH inhibition activity (40.62%) and FRAP values (0.661 mg GAE/g) were found with the BS3 sample compared to all other treatments (Figure 2(b)). The same thing was noticed with salad cream, where the addition of DMP caused a significant increase in the contents of TP and TF and in the antioxidant activity, and the higher TP (5.60 mg GA/g), TF (180.19 *μ*g/mL), and antioxidant activity (17.42% DPPH scavenging activity and FRAP values (0.431 mg GA/g)) were observed with SC3 sample (Figure 2(c)). These increases in TP, TF, and antioxidant activity are due to the dried mushroom powder which has a higher content of total phenolic, total flavonoid, and antioxidant activity as indicated in Table 4. These findings are in agreement with those reported by Arora et al. [67] who observed an increase in % DPPH and total phenolic after incorporation of mushroom flour in instant noodles in comparison with noodles made from 100% wheat flour. Also, Olawuyi and Lee [68] stated that the total phenolic and antioxidant activity in muffin samples were increased with the increase in mushroom flour level. Previous studies suggested that the various phenolic compounds are responsible for quenching the different types of free radicals [69]. The effect of antioxidant components on DPPH radical scavenging activities is due to their ability to hydrogen-donating [70].

### 3.7. Color Measurement of Waffles, Breadsticks, and Salad Cream

Color is one of the most important sensory characteristics that play a vital role in consumer choice and acceptance and preference for any food product. The color parameters (lightness, redness, and yellowness) of the waffles, breadsticks, and salad cream samples are illustrated in Table 7. In the waffle samples, it was obvious that the highest value of lightness (58.46) was found with the W2 sample followed by the W3 sample (58.16); meanwhile, the lowest lightness value (55.27) was obtained with the W0 sample. Otherwise, the highest redness value (17.42) was found in the W0 sample in comparison with the samples containing DMP (W1, W2, and W3). The yellowness value in W2 (39.31) was higher than other treatments and control sample but not statistically significant (*P* > 0.05).

The data in Table 7 show that the addition of DMP caused an increase in the lightness value of breadstick samples which ranged from 64.83 to 70.28 compared to the BS0 sample (59.57). A higher increment of lightness value was observed in BS1 compared with the control sample (BS0). Meanwhile, redness value was slightly increased only with the BS3 sample (17.45) compared with the control sample which recorded the lowest value (17.38) of redness. A higher yellowness value was observed with the BS3 sample compared with all other treatments.

For salad cream product, it could be observed that there were a significant increase in lightness (*L*∗) and redness (*a*∗) values with the increase in the MDP amount in the formula. The highest lightness and redness values were observed in the SC3 sample, while the highest yellowness value was found in the SC2 sample compared with other treatments. These findings might be because DMP was a good source of polyphenol compounds and flavonoids as shown in Table 4. The data obtained in the present study are in agreement with those reported by Losoya-Sifuentes et al. [56] who found that bread fortified with mushroom powder showed a significant increase (*P* ≤ 0.05) in the redness and yellowness values. This may be due to chemical reactions that occur during baking, between the phenolic compounds, the sugars, and proteins contained in the mushroom, thus altering the final color of the product. On the contrary, Salehi et al. [71] reported that the lightness and redness values decreased with increased levels of mushroom flour in cakes. This may be due to the fact that the Maillard reaction also plays a major role in color formation which occurs during baking [72].

### 3.8. Texture Profile Analysis of Waffles, Breadsticks, and Salad Cream

The texture is one of the criteria that determine the quality of the product. The texture properties (hardness, resilience, chewiness, gumminess, cohesiveness, and springiness) of the waffles, breadsticks, and salad cream samples are presented in Table 8. Results showed that the hardness, gumminess, and chewiness of waffle samples significantly (*P* < 0.05) decreased with increasing DMP levels in formulas, while springiness, cohesiveness, and resilience values significantly (*P* < 0.05) increased with the increase in DMP addition. On the other hand, the W0 (control sample) was the highest in hardness, gumminess, and chewiness.

The same thing was observed with breadsticks, where the hardness and springiness values were significantly (*P* < 0.05) decreased with increasing DMP levels. On the other side, with the increase of DMP, the cohesiveness, gumminess, and chewiness values were increased. Control sample (BS0) recorded higher values in hardness and springiness compared to all other treatments containing DMP. As for the salad cream, it was observed that the hardness, chewiness, and gumminess significantly increased with the addition of DMP, while the resilience and cohesiveness values significantly decreased in comparison with the control sample. The control sample (SC0) was the lowest in hardness, springiness, chewiness, and gumminess values in comparison with the DMP samples.

The decrease in hardness is attributed to the destruction of the gluten network [73]. Moreover, the addition of mushroom flour led to higher water content in the formulas, and the presence of protein and fiber in mushroom helped to bond water-reducing hardness [74]. Studies have indicated that mushroom starch contains 56.7% of amylose and 43.3% of amylopectin. Amylose has been associated with hardness and chewiness, while amylopectin is linked to cohesiveness and springiness of products [75]. Cerón-Guevara et al. [74] observed a decrease in hardness, gumminess, cohesiveness, chewiness, and springiness values of frankfurter sausages with increasing the ratio of mushroom flour. Also, Zhang et al. [76] stated that the drying of mushroom caused to textural changes like an increase in hardness and mastication and a decrease in cohesiveness and elasticity, in addition to browning, and the development of unappealing flavor.

### 3.9. Sensory Evaluation of Waffles, Breadsticks, and Salad Cream

#### 3.9.1. Waffles

**Figure 3:**
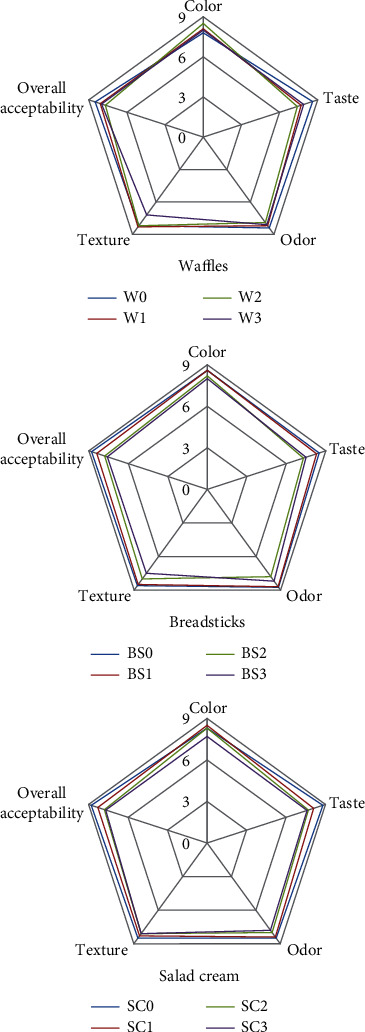
Sensory evaluation of waffles, breadsticks, and salad cream.

The sensory evaluation of the waffles is given in Figure 3. There were no significant (*P* > 0.05) differences in the color, odor, taste, texture, and overall acceptability scores among the control sample (W0) and samples containing different concentrations of DMP, except W2. The change in the taste of the waffles containing DMP compared to the control sample may be attributed to the presence of amino acids in mushroom such as glutamic and aspartic acid [77].

#### 3.9.2. Breadsticks

The results of the sensory evaluation of the breadsticks containing different amounts of DMP are given in Figure 3. The findings indicated that the taste, odor, texture, and overall acceptability scores were significantly (*P* < 0.05) decreased with increasing in DMP amount (BS2 and BS3 samples) but no significant difference between the BS1 sample and control sample (BS0). No significant (*P* > 0.05) differences in color scores among all treatments. These results agreed well with those reported by Ndungù et al. [78] who found that when increasing mushroom flour, the color, taste, texture, aroma, and overall acceptability were significantly (*P* < 0.05) decreased in the composite bread. Also, it recommended the use of mushroom must not exceed 5% in making bread so as not to affect the sensory characteristics of the bread.

#### 3.9.3. Salad Cream

The results in Figure 3 show the sensory evaluation of salad cream. It could be observed that the taste and overall acceptability scores of all samples containing DMP were significantly (*P* < 0.05) decreased compared with the control sample. No significant (*P* > 0.05) differences were found in the color, odor, and texture scores between SC0 (control sample) and salad cream samples containing DMP. Srivastava et al. [57] observed no significant (*P* > 0.05) differences in color and appearance, body or thickness, aroma, or taste of soup up to 30% level of incorporation of mushroom powder.

## 4. Conclusion

This study is aimed at using DMP after exposure to sunlight as a source of vitamin D in some food products such as waffles, breadsticks, and salad cream. The exposure of mushroom to sunlight increased their contents of vitamin D. Also, the addition of DMP to food products increased the contents of protein, fat, and vitamin D in these products, while the carbohydrate content was decreased. The DMP improved the antioxidant activity of food products. It was found that the intake of 100 g of waffles, breadsticks, and salad cream recovered 120, 103, and 82% of the recommended daily allowances of vitamin D, respectively. This study recommended the use of DMP (by a ratio of 3%) in fortifying food products in order to meet the recommended daily allowances of vitamin D.

## Figures and Tables

**Table 1:** Ingredients used for the preparation of waffles, breadsticks, and salad cream.

Ingredients	Formulas of products			
Waffles	W0 (control)	W1 (1% DMP)	W2 (2% DMP)	W3 (3% DMP)
Wheat flour	31.75	30.75	29.75	28.75
DMP	0	1	2	3
Egg	15.87	15.87	15.87	15.87
Sugar	22.22	22.22	22.22	22.22
Salt	0.64	0.64	0.64	0.64
Liquid milk	28.57	28.57	28.57	28.57
Vanilla	0.95	0.95	0.95	0.95

Breadsticks	BS0 (control)	BS1 (1% DMP)	BS2 (2% DMP)	BS3 (3% DMP)

Wheat flour	83.6	82.6	81.6	80.6
DMP	0	1	2	3
Salt	0.4	0.4	0.4	0.4
Yeast	0.5	0.5	0.5	0.5
Sugar	6.7	6.7	6.7	6.7
Corn oil	8.4	8.4	8.4	8.4
Spice	0.4	0.4	0.4	0.4

Salad cream	SC0 (control)	SC1 (1% DMP)	SC2 (2% DMP)	SC3 (3% DMP)

Wheat flour	45	44	43	42
DMP	0	1	2	3
Corn oil	20	20	20	20
Sugar	6	6	6	6
Vinegar	5	5	5	5
Spice	3	3	3	3
Salt	7	7	7	7
Liquid milk	7	7	7	7
Water	7	7	7	7

DMP: Dehydrated mushroom powder.

**Table 2:** Effect of exposure to sunlight on the formation of vitamins D_2_ and D_3_ in fresh mushroom.

Mushroom	V.D_2_ (*μ*g/100 g)	V.D_3_ (*μ*g/100 g)
Not exposed to sunlight	30.03 ± 1.52^c^	62.81 ± 1.51^c^
Exposed to sunlight/60 min	77.61 ± 1.41^a^	70.80 ± 1.70^b^
Exposed to sunlight/120 min	44.61 ± 1.71^b^	99.54 ± 1.46^a^

Results are reported as mean ± SD of triplicate analysis. Means with the same letters are not significantly different (*P* < 0.05) in the same column.

**Table 3:** Chemical composition, bioactive compounds, and antioxidant activity of fresh oyster mushroom (not exposed) and exposed to sunlight for 60 min.

Physical property	Mushroom not exposed to sunlight	Mushroom exposed to sunlight
Chemical composition		
Moisture (%)	91.13 ± 0.23^a^	91.04 ± 0.63^a^
Protein (%)	2.37 ± 0.029^a^	2.39 ± 0.03^a^
Fat (%)	0.163 ± 0.006^a^	0.165 ± 0.011^a^
Ash (%)	0.459 ± 0.021^a^	0.463 ± 0.018^a^
Fiber (%)	0.560 ± 0.015^a^	0.565 ± 0.017^a^
NFE (%)	5.32 ± 0.020^a^	5.37 ± 0.019^a^

Bioactive compounds		

Total phenolic (mg GA/g)	5.28 ± 0.37^b^	7.00 ± 0.17^a^
Total flavonoid (*μ*g RE/mL)	6.90 ± 0.99^b^	18.26 ± 0.80^a^

Antioxidant activity		

DPPH (%)	65.88 ± 0.60^b^	78.74 ± 1.83^a^
FRAP (mg GAE/g)	0.327 ± 0.022^b^	0.390 ± 0.013^a^

Results are reported as mean ± SD of triplicate analysis. Means with the same letters are not significantly different (*P* < 0.05) in the same column. RE: rutin equivalent; GAE: gallic acid equivalent; NEF: carbohydrates; FRAP: ferric reducing antioxidant power; DPPH: 1,1-diphenyl-2-picrylhydrazyl.

**Table 4:** Chemical composition, mineral content, vitamin D content, bioactive compounds, and antioxidant activity of dry mushroom powder (DMP) exposed to sunlight (60 min).

Physical property	Dry mushroom powder (DMP)
Chemical composition	
Moisture (%)	11.13 ± 0.11
Protein (%)	26.71 ± 0.48
Fat (%)	1.84 ± 0.02
Ash (%)	5.17 ± 0.01
Fiber (%)	5.19 ± 0.03
NFE (%)	49.96 ± 0.57

Minerals (mg/100 g)	
Na	239.08 ± 3.20
K	1213.02 ± 0.02
Ca	106.41 ± 3.60
Fe	6.98 ± 0.10
Zn	13.27 ± 0.21
P	912.50 ± 0.0995
Mn	2.43 ± 0.12
Mg	13.18 ± 0.13

Vitamin D (*μ*g/100 g powder)	
V.D_2_	889 ± 3.01
V.D_3_	811 ± 3.44

Bioactive compounds	
Total phenolic (mg GA/g)	6.02 ± 0.33
Total flavonoid (*μ*g RE/mL)	86.29 ± 2.58

Antioxidant activity	
DPPH (%)	90.45 ± 3.01
FRAP (mg GAE/g)	0.420 ± 0.015

RE: rutin equivalent; GAE: gallic acid equivalent; NEF: carbohydrates; FRAP: ferric reducing antioxidant power; DPPH: 1,1-diphenyl-2-picrylhydrazyl.

**Table 5:** The chemical composition of waffles, breadsticks, and salad cream.

Products	Moisture (%)	Protein (%)	Fat (%)	Ash (%)	NFE (%)
Waffles					
W0 (control)	50.16 ± 0.04^a^	4.50 ± 0.03^c^	6.21 ± 0.01^a^	0.694 ± 0.014^b^	38.44 ± 0.09^a^
W1 (1% DMP)	49.94 ± 0.17^a^	4.76 ± 0.07^c^	6.22 ± 0.01^a^	0.720 ± 0.002^a^	38.37 ± 0.14^a^
W2 (2% DMP)	49.93 ± 0.44^a^	5.33 ± 0.26^b^	6.23 ± 0.01^a^	0.728 ± 0.006^a^	37.79 ± 0.38^a^
W3 (3% DMP)	49.55 ± 0.66^a^	5.74 ± 0.12^a^	6.26 ± 0.11^a^	0.734 ± 0.006^a^	37.71 ± 0.73^a^

Breadsticks					

BS0 (control)	28.37 ± 0.40^a^	15.01 ± 0.58^b^	3.53 ± 0.10^a^	1.88 ± 0.10^b^	51.20 ± 1.19^a^
BS1 (1% DMP)	28.42 ± 0.19^a^	15.47 ± 0.70^ab^	3.53 ± 0.09^a^	1.96 ± 0.01^b^	50.63 ± 0.98^a^
BS2 (2% DMP)	27.11 ± 0.10^b^	15.96 ± 0.25^ab^	3.59 ± 0.16^a^	2.12 ± 0.21^b^	51.23 ± 0.21^a^
BS3 (3% DMP)	27.26 ± 0.10^b^	16.22 ± 0.39^a^	3.64 ± 0.03^a^	2.57 ± 0.13^a^	50.31 ± 1.29^a^

Salad cream					

SC0 (control)	47.88 ± 0.20^a^	10.05 ± 0.39^b^	19.90 ± 1.36^a^	1.50 ± 0.15^c^	20.67 ± 0.17^a^
SC1 (1% DMP)	47.96 ± 0.07^a^	10.18 ± 0.22^ab^	20.13 ± 0.88^a^	2.08 ± 0.04^b^	19.64 ± 0.11^ab^
SC2 (2% DMP)	48.00 ± 1.02^a^	10.67 ± 0.48^ab^	20.23 ± 0.19^a^	2.29 ± 0.25^ab^	18.81 ± 0.10^ab^
SC3 (3% DMP)	48.05 ± 0.28^a^	10.86 ± 0.27^a^	20.31 ± 0.69^a^	2.45 ± 0.14^a^	18.33 ± 0.70^b^

DMP: Oyster mushroom powder. Results are reported as mean ± SD of triplicate analysis. Means with the same letters are not significantly different (*P* < 0.05) in the same column.

**Table 6:** Vitamin D_2_ and D_3_ contents (*μ*g) and % of the recommended daily allowances (RDA) from each 100 g waffles, breadsticks, and salad cream.

Products	V.D (*μ*g/100 g product)		RDA/life stage group (years)			
			Children (1-8 Y)	Males and females (9-70 Y)	Males and females (> 70 Y)	Pregnancy and lactation
			600 IU (15 *μ*g)	600 IU (15 *μ*g)	800 IU (20 *μ*g)	600 IU (15 *μ*g)
Waffles						
W0 (control)	V.D_2_	0.995 ± 0.106^d^	6.63	6.63	4.97	6.63
	V.D_3_	1.480 ± 0.090^d^	9.87	9.87	7.40	9.87

W1 (1% DMP)	V.D_2_	5.152 ± 0.052^c^	34.35	34.35	25.76	34.35
	V.D_3_	4.825 ± 0.225^c^	32.16	32.16	24.12	32.16

W2 (2% DMP)	V.D_2_	9.919 ± 0.160^b^	66.12	66.12	49.59	66.12
	V.D_3_	8.592 ± 0.188^b^	57.28	57.28	42.96	57.28

W3 (3% DMP)	V.D_2_	15.458 ± 0.21^a^	103.06	103.06	77.29	103.06
	V.D_3_	12.472 ± 0.190^a^	83.15	83.15	62.36	83.15

Breadsticks						

BS0 (control)	V.D_2_	0.569 ± 0.014^d^	3.79	3.79	2.84	3.79
	V.D_3_	0.422 ± 0.068^d^	2.81	2.81	2.11	2.81

BS1 (1% DMP)	V.D_2_	4.119 ± 0.120^c^	27.46	27.46	20.60	27.46
	V.D_3_	3.059 ± 0.240^c^	20.39	20.39	15.29	20.39

BS2 (2% DMP)	V.D_2_	8.185 ± 0.166^b^	54.57	54.57	40.92	54.57
	V.D_3_	6.078 ± 0.152^b^	40.52	40.52	30.39	40.52

BS3 (3% DMP)	V.D_2_	12.352 ± 0.171^a^	82.35	82.35	61.76	82.35
	V.D_3_	9.168 ± 0.032^a^	61.12	61.12	45.84	61.12

Salad cream						

SC0 (control)	V.D_2_	0.550 ± 0.014^d^	3.66	3.66	2.75	3.66
	V.D_3_	0.608 ± 0.024^d^	4.05	4.05	3.03	4.05

SC1 (1% DMP)	V.D_2_	6.302 ± 0.198^c^	42.01	42.01	31.51	42.01
	V.D_3_	4.877 ± 0.224^c^	32.51	32.51	24.38	32.51

SC2 (2% DMP)	V.D_2_	12.571 ± 0.247^b^	83.81	83.81	62.85	83.81
	V.D_3_	9.522 ± 0.154^b^	63.48	63.48	47.61	63.48
SC3 (3% DMP)	V.D_2_	18.122 ± 0.205^a^	120.81	120.81	90.61	120.81
	V.D_3_	13.647 ± 0.224^a^	90.98	90.98	68.23	90.98

DMP: Oyster mushroom powder. Results are reported as mean ± SD of triplicate analysis. Means are significantly different (*P* < 0.05) in the same column.

**Table 7:** Color measurement of waffles, breadsticks, and salad cream.

Products	Lightness (*L*^∗^)	Redness (*a*^∗^)	Yellowness (*b*^∗^)
Waffles			

W0 (control)	55.27 ± 1.27^b^	17.42 ± 1.58^a^	34.21 ± 1.69^b^
W1 (1%DMP)	56.87 ± 2.34^ab^	16.60 ± 1.30^a^	37.19 ± 1.19^a^
W2 (2% DMP)	58.46 ± 1.19^a^	16.06 ± 1.03^a^	39.31 ± 1.9^a^
W3 (3% DMP)	58.16 ± 1.01^ab^	16.16 ± 1.02^a^	38.86 ± 1.14^a^

Breadsticks			

BS0 (control)	59.56 ± 1.01^c^	17.38 ± 1.84^a^	37.33 ± 0.93^b^
BS1 (1%DMP)	70.28 ± 1.62^a^	13.95 ± 1.26^b^	37.58 ± 1.06^ab^
BS2 (2% DMP)	68.73 ± 1.08^a^	14.48 ± 0.60^b^	36.17 ± 1.03^b^
BS3 (3% DMP)	64.83 ± 1.33^b^	17.45 ± 0.57^a^	39.50 ± 1.10^a^

Salad cream			

SC0 (control)	52.39 ± 1.61^c^	0.28 ± 0.014^d^	9.36 ± 0.86^c^
SC1 (1%DMP)	68.37 ± 0.85^a^	1.89 ± 0.070^b^	18.93 ± 0.91^b^
SC2 (2% DMP)	65.24 ± 1.24^b^	1.49 ± 0.11^c^	22.36 ± 1.65^a^
SC3 (3% DMP)	69.92 ± 1.32^a^	2.65 ± 0.15^a^	17.46 ± 0.93^b^

DMP: oyster mushroom powder. Results are reported as mean ± SD of triplicate analysis. Means with the same letters are not significantly different (*P* < 0.05) in the same column.

**Table 8:** Texture profile analysis of waffles, breadsticks, and salad cream.

Products	Hardness (g)	Resilience	Cohesiveness	Springiness (mm)	Gumminess (g)	Chewiness (mJ)
Waffles						
W0 (control)	9027 ± 22.50^a^	0.06 ± 0.006^c^	0.15 ± 0.006^b^	0.98 ± 0.07^b^	1363 ± 7.1^a^	13.1 ± 0.31^b^
W1 (1%DMP)	3188 ± 12.50^b^	0.50 ± 0.061^a^	0.25 ± 0.071^b^	1.03 ± 0.08^b^	1181 ± 9.2^b^	19.9 ± 0.51^a^
W2 (2% DMP)	1517 ± 15.50^c^	0.34 ± 0.026^b^	0.78 ± 0.105^a^	1.72 ± 0.03^a^	801 ± 6.1^c^	8.7 ± 0.41^c^
W3 (3% DMP)	576 ± 5.51^d^	0.31 ± 0.021^b^	0.86 ± 0.080^a^	1.79 ± 0.11^a^	493 ± 8.0^d^	8.1 ± 0.51^c^
Breadsticks						
BS0 (control)	7468 ± 8.51^a^	0.04 ± 0.004^b^	0.02 ± 0.001^c^	0.54 ± 0.02^b^	158 ± 3.5^d^	0.8 ± 0.06^c^
BS1 (1%DMP)	6086 ± 5.51^b^	0.02 ± 0.001^c^	0.09 ± 0.005^a^	1.09 ± 0.09^a^	527 ± 2.52^a^	5.2 ± 0.21^a^
BS2 (2% DMP)	5461 ± 6.51^c^	0.01 ± 0.003^d^	0.01 ± 0.003^d^	0.40 ± 0.02^c^	370 ± 1.48^b^	1.1 ± 0.006^b^
BS3 (3% DMP)	3240 ± 5.51^d^	0.05 ± 0.003^a^	0.07 ± 0.003^b^	0.49 ± 0.03^bc^	231 ± 2.47^c^	0.1 ± 0.095^d^
Salad cream						
SC0 (control)	22.0 ± 1.05^d^	0.35 ± 0.02^a^	0.81 ± 0.04^a^	3.54 ± 0.17^a^	18.0 ± 0.95^d^	0.6 ± 0.03^b^
SC1 (1%DMP)	28.0 ± 2.04^c^	0.24 ± 0.02^b^	0.78 ± 0.02^a^	3.47 ± 0.20^a^	22.0 ± 1.05^c^	0.7 ± 0.02^b^
SC2 (2% DMP)	38.0 ± 1.05^b^	0.15 ± 0.01^c^	0.69 ± 0.02^b^	2.72 ± 0.18^b^	32.0 ± 0.99^b^	0.3 ± 0.01^c^
SC3 (3% DMP)	51.1 ± 1.15^a^	0.13 ± 0.02^c^	0.80 ± 0.03^a^	3.64 ± 0.09^a^	41.0 ± 2.95^a^	1.5 ± 0.11^a^

DMP: oyster mushroom powder. Results are reported as mean ± SD of triplicate analysis. Means with the same letters are not significantly different (*P* < 0.05) in the same column.

## Data Availability

The datasets generated during and/or analyzed during the current study are available from the corresponding author on reasonable request.
